# Retaining women in a prenatal care randomized controlled trial in Canada: implications for program planning

**DOI:** 10.1186/1471-2458-7-148

**Published:** 2007-07-06

**Authors:** Suzanne C Tough, Jodi E Siever, David W Johnston

**Affiliations:** 1 Department of Paediatrics, University of Calgary, Calgary, Alberta, T3B 6A8, Canada; 2 Department of Community Health Sciences, University of Calgary, Calgary, Alberta, T2N 4N1, Canada; 3 Decision Support Research Team, Calgary Health Region, Calgary, Alberta, T3B 6A8, Canada

## Abstract

**Background:**

Challenges to retention in prenatal care seem to exist under both universal systems of care, as in Canada, and non-universal systems of care, as in the United States. However, among populations being served by a system of publicly funded health care, the barriers are less well understood and universal uptake of prenatal services has not been realized. Determining the characteristics of women who dropped out of a prenatal care randomized controlled trial can help identify those who may need alternate retention and service approaches.

**Methods:**

In this study, pregnant women were randomized to: a) current standard of care; b) 'a' plus nursing support; or c) 'b' plus a paraprofessional home visitor. 16% of 2,015 women did not complete all three telephone interviews (197 dropped out and 124 became unreachable). Responders were compared to non-responders on demographics, lifestyle, psychosocial factors, and life events using chi-squared tests. Logistic regression models were constructed using stepwise logistic regression to determine the probability of not completing the prenatal program.

**Results:**

Completion rates did not differ by intervention. In comparison to responders, non-responders were more likely to be younger, less educated, have lower incomes, smoke, have low social support, have a history of depression, and have separated or divorced parents (all p < 0.05). Unreachable women were more likely to be single, use drugs, report distress and adverse life events (all p < 0.05). Non-Caucasian women were more likely to drop out (p = 0.002). Logistic regression modeling indicated that independent key risk factors for dropping out were: less than high school education, separated or divorced parents, lower social support, and being non-Caucasian. Pregnant women who were single/separated/divorced, less than 25 years old, had less than high school education, earned less than $40,000 in annual household income, and/or smoked had greater odds of becoming unreachable at some point during pregnancy and not completing the study.

**Conclusion:**

Women at risk due to lifestyle and challenging circumstances were difficult to retain in a prenatal care study, regardless of the intervention. For women with complex health, lifestyle and social issues, lack of retention may reflect incongruence between their needs and the program.

**Trial registration:**

Current Controlled Trials ISRCTN64070727

## Background

Randomized intervention trials provide high level evidence for program planning, but attrition of participants is a common research challenge which can reduce the generalizability and potential relevance of study findings, as well as contribute to bias in results [1,2]. Furthermore, the impact of an intervention is often based on attendance and engagement (intervention intensity). Intervention studies and programs typically incorporate retention strategies to ensure program goals are achieved and to prevent attrition of participants. Determining the characteristics of participants who do not complete prenatal intervention studies and programs can help identify gaps in program generalizability and identify who may require different services and retention strategies. This paper compares the characteristics of women who completed a community-based randomized controlled trial of supplementary prenatal care in Canada to those who did not. This information can be used by program planners, clinicians and researchers to develop strategies that will improve rates of engagement and retention in prenatal programs, with the ultimate goal of improved prenatal and maternal health and well being.

The primary purpose of prenatal care is to support a healthy pregnancy and delivery through medical care, support, health promotion, screening, and referral and access to appropriate community resources [3]. Through prenatal care there is an opportunity to engage women in programs and resources, such as food security, smoking cessation or social support, that improve the chances of a healthy pregnancy. The prenatal period is also an opportune time to support women in behaviour changes which could improve both the prenatal and early postpartum experience (e.g. parenting, connection to community) [3].

Attrition rates in prenatal care programs range from 4% to 41%, suggesting that in some cases, retention is a substantial barrier to achieving program objectives [4-7]. The failure to retain women in prenatal programs may reflect weak therapeutic alliance [8,9], lack of peer support, lack of perceived benefit or need of support, high levels of daily "life hassles" [10], fear of "the system", logistical challenges and/or residential instability during pregnancy [11]. Strategies used to improve prenatal care program attendance in the past have included provision of baby items, department store gift certificates, and free transportation to clinic appointments [4]. Other strategies to maximize retention and participation in prenatal care include earlier initiation of care, more frequent contact with providers, gift incentives for completing each component of a program, multiple contact sources to help locate patients, and using focus groups to ensure programs are culturally sensitive [4].

Challenges to retention in prenatal care seem to exist under both universal systems of care, as in Canada, and non-universal systems of care, as in the United States [11]. Data from the United States suggests that women at risk of both poor birth outcomes and non-use or under-use of prenatal services are more likely to be non-Caucasian, have less than high school education, use Medicaid support, be single parents, be of low income, have low social support, smoke, use street drugs, and have their first birth before the age of 20 [4,10,12,13]. Evidence from European countries with universal health care systems suggests that late or inadequate care increases with parity and extremes of maternal age [14]. Furthermore, even under a system of universal care, women with low education, young maternal age, single marital status, and low levels of social support are most likely to report dissatisfaction, and potentially reduced compliance, with prenatal care [15-17].

The suboptimal uptake of community based prenatal resources in Calgary combined with local client feedback that prenatal services were not received early enough in pregnancy [18] led to questions about optimal administration and planning of prenatal care and services, under a system of universal health care where midwifery is not routinely funded. However, evidence of the effectiveness of supplementary prenatal support from nurses and home visitors within the context of low risk community based prenatal care was equivocal, and there was uncertainty about the generalizability of findings from high risk populations [19-25]. Thus, this Canadian randomized controlled trial was conducted to support evidence-informed program planning by investigating the impact of supplementary prenatal care on referral and access to appropriate resources. The primary results of this trial have been published elsewhere [26]. In summary, women who benefited from supplementary prenatal support provided by either a nurse or nurse plus home visitor had the following characteristics: they were delivering their first live born child, smoked, had a lower income, were of young maternal age, were non-Caucasian, had experienced abuse in the past, and were more unwilling to maintain, nurture, or use social supports. However, women who received supplementary prenatal support were just as likely to complete the study as women who received the current standard of care. Thus, this paper seeks to determine which women were not retained in this study to develop strategies that may help retain this population and consequently optimize maternal well being and pregnancy outcomes. Because this study achieved a recruitment rate of 71.1% among those eligible to participate, a completion rate of 82.9%, and similar completion rates across condition, there is an ideal opportunity to understand where prenatal services can be further improved and for whom.

## Methods

Pregnant women at low medical risk who sought services provided by family physicians at one of three participating maternity clinics in a Canadian city were recruited into the intervention study between April 2001 and July 2004. Women were contacted after booking their first prenatal appointment. After informed consent and the initial interview, 1,737 women were randomized to one of three groups: a) current standard of physician care; b) current standard of care plus consultation with a nurse trained to provide prenatal support; or c) 'b' plus consultation with a paraprofessional home visitor trained to provide non-medical prenatal support.

Participants completed three telephone interviews (first trimester, 32 to 34 weeks gestation and six to eight weeks post-delivery) related to demographics, life events, pregnancy related issues and resource utilization. The interviews were conducted in English as well as several other languages including Urdu, Punjabi, French, Cantonese, Mandarin, and Arabic. Many retention strategies were included in this study, including multiple contact sources to help locate participants, repeated phone calls to reach women, having the nurse meet women in a convenient location (primarily the maternity clinic but also the home, coffee shops, and work places), and employing multi-lingual nurses and home visitors. In this research context, ethics restrictions precluded the provision of gift incentives.

This research was carried out in compliance with the Helsinki Declaration. All participants provided informed consent and the study received ethical approval from the Conjoint Medical Bioethics Committee of the University of Calgary and Calgary Health Region (ref#15763). Further details of the study methodology are available elsewhere [26,27].

**Figure 1:**
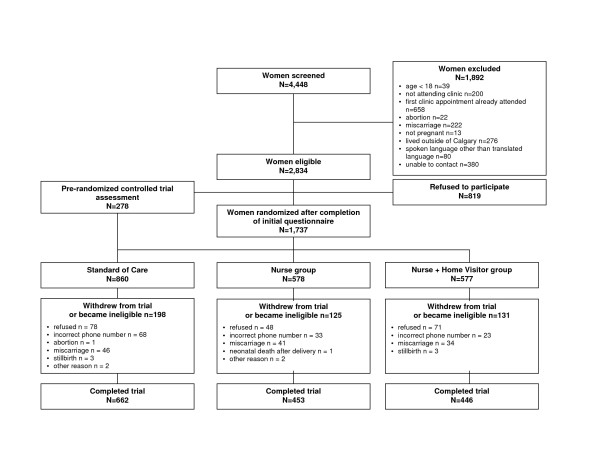
Study flowchart mapping recruitment and randomization of participating women to Control, Nurse, or Nurse plus Home Visitor study groups.

The purpose of this analysis was to determine if women who did not complete the third interview differed in a systematic way from women who completed all three interviews. Participants were classified as "drop-outs" if they withdrew from the study anytime before completion of the third interview. Women who could not be contacted by telephone due to a change in phone number prior to completion of the third interview were considered "unreachable." Participants considered to be "responders" completed all three interviews. Women were excluded from the analysis if they had a miscarriage, abortion, still birth, or neonatal death (n = 133) (Figure 1). In this analysis, 278 women from a pre-randomized controlled trial phase were also included in the standard of care group. These women were recruited over a 12 week period prior to the randomized controlled trial to assess changes in clinic practice resulting from the introduction of the intervention. Aside from being recruited earlier, these women proceeded as per the standard of care study protocol. Women who refused to participate in the pre-randomized controlled trial phase were not included in further assessments.

Responders, drop-outs, and unreachable women were compared at the bi-variate level with respect to socio-demographic characteristics, obstetrical history, lifestyle choices, psychosocial health, and life events. Ethnicity/race was self reported based on the question "How would you best describe your ethnic origin/race?" and then categorized into Caucasian and Non-Caucasian. For this project, non-Caucasian was defined to include Latin American, Aboriginal, South Asian, South East Asian, West Asian/Arab, African American, Chinese, Filipino, Greek, Italian, Japanese or Korean. Chi-squared tests were used for conducting the analysis, and a p-value less than or equal to 0.05 was considered statistically significant. Variables found to be significant in the bivariate analysis were then eligible for multivariable modeling. Two logistic regression models, one for drop-outs and one for unreachable women, were constructed using stepwise logistic regression with the forward selection approach to determine the probability of not completing the prenatal program. All statistical analyses were performed using Stata 8/SE version 8.2 [28].

## Results

A total of 1,561 (82.9%) women responded to all three interviews, 197 (9.8%) women dropped out and 124 (6.2%) women became unreachable. The proportion of women who dropped out or became unreachable was similar across study groups. Among women considered to be at higher risk for marginalization (as defined by age <25, single, <high school education, <$40,000 annual income), 74.6% responded to all three interviews as compared to 88.4% of lower risk women.

Women who did not complete the study (drop-out or unreachable) differed significantly from responders for several socio-demographic characteristics (Table 1). Young maternal age, lower levels of education, and low income increased the likelihood of either dropping out or becoming unreachable (Table 1). Non-Caucasian women were significantly more likely to drop-out (Table 1). Women without partners were significantly more likely to become unreachable by the post partum interview (Table 1).

**Table 1:** Sociodemographic characteristics, lifestyle choices, and food security of women who completed the prenatal care trial, compared to women who dropped out or became unreachable

	**Responder** **N = 1561** **n (%)**	**Drop out** **N = 197** **n (%)**	**Unreachable** **N = 124** **n (%)**	**Responder vs. Drop out Unadjusted Odds Ratio (95% CI)**	**p-value**	**Responder vs. Unreachable Unadjusted Odds Ratio (95% CI)**	**p-value**
**Sociodemographic characteristics**							
Maternal age							
18–24	289 (18.6)	51 (26.6)	65 (52.9)	1.59 (1.13,2.24)	0.008	4.92 (3.38,7.17)	<0.00
25+	1269 (81.5)	141 (73.4)	58 (47.2)	Reference		Reference	1
Marital status							
Married/common law	1448 (92.8)	181 (92.4)	96 (77.4)	Reference	0.809	Reference	<0.00
Not married/separated/divorced	112 (7.2)	15 (7.6)	28 (22.6)	1.07 (0.61,1.88)		3.77 (2.37,5.99)	1
Has spouse/partner							
Yes	1533 (98.2)	193 (98.5)	115 (92.7)	Reference	0.792	Reference	<0.00
No	28 (1.8)	3 (1.5)	9 (7.3)	0.85 (0.26,2.83)		4.28 (1.97, 9.30)	1
Ethnicity							
Caucasian	1183 (75.78)	129 (65.8)	87 (70.2)	Reference	0.002	Reference	0.162
Not Caucasian	378 (24.2)	67 (34.2)	37 (29.8)	1.62 (1.18,2.23)		1.33 (0.89,1.99)	
Education							
Less than high school	116 (7.5)	31 (15.8)	37 (29.8)	2.44 (1.58,3.78)	<0.00	7.56 (4.74,12.07)	<0.00
High school	279 (17.9)	38 (19.4)	38 (30.7)	1.25 (0.85,1.83)	1	3.23 (2.07,5.03)	1
University/college/business school	1162 (74.6)	127 (64.8)	49 (39.5)	Reference		Reference	
Income							
< $40000	109 (9.0)	59 (15.5)	59 (54.1)	1.87 (1.33, 2.63)	<0.00	4.08 (2.74, 6.06)	<0.00
≥ $40000	1109 (91.0)	321 (84.5)	50 (45.9)	Reference	1	Reference	1
**Lifestyle Choices**							
Smoking*							
Not at all	1190 (76.2)	136 (69.4)	58 (47.2)	Reference	0.007	Reference	<0.00
Occasionally	73 (4.7)	5 (2.6)	9 (7.3)	0.60 (0.24,1.51)		2.53 (1.21,5.31)	1
Daily	298 (19.1)	55 (28.1)	56 (45.5)	1.61 (1.15,2.26)		3.86 (2.61,5.69)	
Alcohol*							
Rarely (1/wk to less than 1/month)	1349 (86.6)	174 (88.3)	104 (83.9)	Reference	0.676	Reference	0.500
Occasionally (2–3/wk)	170 (10.9)	20 (10.2)	15 (12.1)	0.91 (0.56,1.49)		1.14 (0.65,2.01)	
Daily	38 (2.4)	3 (1.5)	5 (4.0)	0.61 (0.18, 2.00)		1.71 (0.66,4.43)	
Street drugs*							
Yes	134 (8.6)	16 (8.1)	28 (22.8)	0.94 (0.55, 1.61)	0.823	3.13 (1.98, 4.95)	<0.00
No	1425 (91.4)	181 (91.9)	95 (77.2)	Reference		Reference	1
**Food Security**							
Food bank*							
Yes	67 (4.3)	19 (9.6)	23 (18.7)	2.38 (1.40,4.05)	0.001	5.12 (3.06,8.58)	<0.00
No	1493 (95.7)	178 (90.4)	100 (81.3)	Reference		Reference	1

*Within 12 months of pregnancy

Women in this study were also compared for lifestyle choices and food security (Table 1). A greater proportion of drop-outs and unreachable women reported daily smoking and the use of a food bank at least once within the twelve months prior to pregnancy (Table 1). Compared to women who responded to all three interviews, a greater proportion of unreachable women reported using recreational street drugs within twelve months of becoming pregnant (Table 1).

Compared to responders, women who dropped out or became unreachable had lower social support and were less willing to use social networks (i.e. higher negative network orientation) (Table 2). In addition, overall distress was higher in women who became unreachable during the study (Table 2). In examining life events, drop-outs and unreachable women were significantly more likely to have separated or divorced parents and a history of depression (Table 2). Unreachable women were significantly more likely to report a history of drug problems, alcohol problems, suicidal thoughts or attempts, and being unemployed for a lengthy amount of time when they wanted to be working (Table 2).

**Table 2:** Psychosocial characteristics and life events of women who completed the prenatal care trial, compared to women who dropped out or became unreachable

	**Responder** **N = 1561** **n (%)**	**Drop out** **N = 197** **n (%)**	**Unreachable** **N = 124** **n (%)**	**Responder vs. Drop out Unadjusted Odds Ratio (95% CI)**	**p-value**	**Responder vs. Unreachable Unadjusted Odds Ratio (95% CI)**	**p-value**
**Psychosocial characteristic**							
Social Support*							
High	1102 (70.7)	115 (59.3)	70 (56.9)	Reference	0.001	Reference	0.001
Low	456 (29.3)	79 (40.7)	53 (43.1)	1.66 (1.22,2.26)		1.83 (1.26,2.66)	
Negative Network Orientation*+							
High	550 (35.2)	92 (46.7)	61 (49.2)	1.61 (1.19,2.17)	0.002	1.78 (1.23,2.57)	0.002
Low	1011 (64.8)	105 (53.3)	63 (50.8)	Reference		Reference	
Self Esteem*							
High	1112 (71.4)	131 (66.5)	79 (63.7)	Reference	0.156	Reference	0.071
Low	446 (28.6)	66 (33.5)	45 (36.3)	1.25 (0.92,1.72)		1.42 (0.97,2.08)	
Total Distress*							
High	1013 (64.9)	123 (62.4)	68 (54.8)	1.11 (0.82,1.51)	0.497	1.52 (1.05,2.20)	0.025
Low	548 (35.1)	74 (37.6)	56 (45.2)	Reference		Reference	
**Life Events**							
History of drug problems							
Yes	54 (3.5)	5 (2.5)	13 (10.5)	0.76 (0.29, 1.84)	0.499	3.27 (1.73, 6.17)	<0.001
No	1507 (96.5)	192 (97.5)	111 (89.5)	Reference		Reference	
History of alcohol problems							
Yes	53 (3.4)	6 (3.1)	10 (8.1)	0.89 (0.38, 2.11)	0.797	2.50 (1.24, 5.04)	0.008
No	1508 (96.6)	191 (96.9)	114 (91.9)	Reference		Reference	
History of unemployed when wanted to work							
Yes	161 (10.3)	26 (13.2)	29 (23.4)	1.32 (0.85, 2.06)	0.217	2.65 (1.70, 4.15)	<0.001
No	1399 (89.7)	171 (86.8)	95 (76.6)	Reference		Reference	
History of depression							
Yes	353 (22.7)	57 (29.1)	47 (38.2)	1.40 (1.00,1.95)	0.046	2.11 (1.43,3.09)	<0.001
No	1204 (77.3)	139 (70.9)	76 (61.8)	Reference		Reference	
History of suicidal thoughts or attempt							
Yes	162 (10.4)	21 (10.7)	21 (17.1)	1.03 (0.64, 1.67)	0.894	1.77 (1.08, 2.91)	0.022
No	1395 (89.6)	175 (89.3)	102 (82.9)	Reference		Reference	
History of parents separated or divorced							
Yes	451 (28.9)	74 (37.6)	60 (48.4)	1.48 (1.09,2.01)	0.012	2.31 (1.59,3.33)	<0.001
No	1109 (71.1)	123 (62.4)	64 (51.6)	Reference		Reference	

*variable dichotomized at top or bottom third of scores

+Women were considered to have negative network orientation if they scored in the top 33^rd ^percent of all network orientation scores from this sample, meaning they were unwilling or reluctant to maintain, nurture, or use those social supports that they had.

Logistic regression modeling indicated that independent risk factors for dropping out included: non-Caucasian, less than high school education, separated or divorced parents, and lower social support (Table 3). Pregnant women who were single, less than 25 years old, had less than high school education, earned less than $40,000 in annual household income, and/or smoked daily prior to pregnancy had greater odds of becoming unreachable at some point during pregnancy and not completing the study (Table 3).

**Table 3:** Logistic regression for women who did not complete the prenatal study

	**Responder** **N = 1561** **n (%)**	**Drop out** **N = 197** **n (%)**	**Adjusted Odds Ratio**	**95% CI**
**Responders vs. Drop-outs**				

Non-Caucasian	378 (24.2)	67 (34.2)	1.55	(1.11, 2.19)
Education < high school	116 (7.5)	31 (15.8)	1.86	(1.17, 2.95)
Parents separated or divorced	451 (28.9)	74 (37.6)	1.47	(1.06, 2.04)
Low social support	456 (29.3)	79 (40.7)	1.43	(1.05, 1.97)

	**Responder** **N = 1561** **n (%)**	**Unreachable** **N = 124** **n (%)**		

**Responders vs. Unreachables**				

Maternal age < 25 years	289 (18.6)	65 (52.9)	2.17	(1.33, 3.52)
Education < high school	116 (7.5)	37 (29.8)	2.60	(1.40, 4.83)
Single/Separated/Divorced	112 (7.2)	28 (22.6)	1.89	(1.09, 3.28)
Income < $40000	109 (9.0)	59 (54.1)	2.04	(1.28, 3.24)
Smoke daily	298 (19.1)	56 (45.5)	1.86	(1.17, 2.93)

## Discussion

With intervention studies and programs that require commitment over time, retention of participants is a common challenge. In this large community-based randomized controlled trial of prenatal care, retention rates exceeded 80% and retention was similar across study groups. However, even under a system of universal care, and with provision of additional support from nurses and home visitors, there were unique populations of women who were not retained in this prenatal care study. In particular, women who were non-Caucasian, with less than high school education, separated or divorced parents, lower social support, who were single, less than 25 years old, earned less than $40,000 in annual household income, and/or smoked daily prior to pregnancy were less likely to complete the study. Others have noted that characteristics of women least likely to complete studies of prenatal care include: African-American, younger, lower education, lower income, higher parity, and/or foreign born [29,30]. Research suggests that barriers to prenatal care among low income women are both financial (e.g. lack of convenient transportation) and psychosocial (e.g. not feeling comfortable as a single woman in a prenatal class attended largely by couples) [11,31]. Furthermore, in addition to language barriers, non-Caucasian women may have prenatal care traditions that differ from western culture, including more peer and family support, home visitation, as well as midwifery [26,32].

To help retain women and ensure that they receive adequate and evidence-based prenatal care, prenatal programs and studies should consider assessing how well their programs are meeting the needs of their clients, including consideration of cultural traditions, psychosocial variables, and lifestyle factors. Establishing health care practices where "at risk" women spend time, providing low or no cost child care, and allowing for flexible appointment scheduling (e.g. evening appointments or drop-in services) could help remove barriers for some women and may reduce attrition. Currently, some grocery stores offer no cost child care while customers shop and also host periodic health assessments, such as blood pressure and bone density assessments. These proprietors may be natural community partners for pilot testing new methods of service delivery. Delivering prenatal care in a group setting may also serve the needs of some women better. Preliminary evidence on such programs indicates high participation and completion rates, a reduction in low birth weight rates, as well as high client satisfaction, suggesting that group prenatal care may be both acceptable and effective at retaining women in care and services [33,34]. Prior to the development of new programs, though, additional input from women less likely to complete prenatal programs may reveal other barriers to service which could be addressed in the design of new programs [11].

Direct incentives for participation in prenatal care may also help retain women in prenatal care programs. Many European countries offer pregnancy allowances, attending prenatal care during paid working hours, protection from job loss during pregnancy and the guarantee of a safe work environment (e.g. chemical free, no heavy lifting) and indeed, among eight European countries, the proportion of women who had less than three prenatal visits ranged from 0.4% to 6% [14]. Consequently, system level changes may also be appropriate mechanisms for improving retention in prenatal care programs.

Participation in health promotion programs such as prenatal care is also contingent upon client perception of benefit [4,35]. When participants who chose to drop out were asked by telephone interviewers for their reason, many indicated they were too busy and did not have the time. This potentially indicates that the benefits of participating in this intervention study were not valued highly enough, recognized or received by some women. Indeed, there may be un-recognized gaps or limitations in services delivery programs which hinders uptake and retention of women with complex lives.

Although the characteristics of women at risk of not being retained in this study were similar to those of women at risk of both poor birth outcomes and non-use or low use of prenatal services under a both universal and non-universal systems of care, the generalization from research to program planning should be evaluated on a site specific basis during program development and pilot testing [4,10,12,13]. A limitation to this analysis was our inability to unequivocally determine who completed the intervention program with the Nurse and/or Home Visitor but did not complete the study interviews. In this analysis, women who received program care but did not complete the study would be included in the non-responder group. Consequently, our non-responders group may have included women who were more similar to responders which would bias our findings towards no difference between groups. This bias, in addition to the potential challenges associated with generalizing study findings to a program setting, should be considered when interpreting the results.

The routine contact with health care providers that occurs during pregnancy provides a critical opportunity to identify and address health and psychosocial needs, and additional support at this time can indeed improve resource utilization. The primary objective of this study was to measure resource use, but on-going follow-up of this cohort will allow for assessment of behaviors, such as breastfeeding and early parenting. Finally, the finding that women lost to follow up had more risk characteristics, including young maternal age, non-Caucasian, and lower education, suggests that a program targeted to women with higher needs may require additional and innovative retention strategies (e.g. day care, transportation support, flexible appointment times) if optimal program effects are to be realized. Based on this study, the Calgary Health Region is undertaking a review of services in a quadrant of the city which has a high proportion of underserved citizens and is also planning to pilot test group prenatal care. The Calgary Health Region may also consider delivering services in non-medical settings and in locations with less restrictive appointment times, in an attempt to reach those who remain at highest risk of not being served.

## Conclusion

Despite significant retention efforts, a higher proportion of women with complex lives and multiple risk factors did not complete the study. For program evaluators and policy makers, asking if an intervention worked is not as helpful as asking how can we implement both universal and targeted programs to improve outcomes for all women. We conclude that, even under a universal system of care and with supplementary prenatal support, optimal birth and early childhood outcomes will not be achieved until programs and resources are implemented which better meet the needs and preferences of all women [12,13]. Program planners should be aware that recruitment and retention strategies will be more effective among women with complex lives and multiple risk factors when tailored to the needs of this clientele.

## Competing interests

The author(s) declare that they have no competing interests.

## Authors' contributions

SCT conceived of the study and supervised all aspects of its implementation. JES completed the analyses. DWJ managed study implementation and data collection. All authors helped to conceptualize ideas, interpret findings, and review and revise drafts of the manuscript. All authors read and approved the final manuscript.

## Pre-publication history

The pre-publication history for this paper can be accessed here:


